# Growth pattern can be used as a new characteristic to predict malignancy in breast cancer

**DOI:** 10.1007/s12282-019-01041-7

**Published:** 2020-01-03

**Authors:** Bin Wang, Lizhe Zhu, Chenyang He, Minghui Tai, Can Zhou, Guanqun Ge, Huimin Zhang, Jianjun He, Ke Wang

**Affiliations:** 1 Department of Breast Surgery, The First Affiliated Hospital of Xi’an Jiaotong University, 277 Yanta Western Road, Xi’ an, 710061 Shaanxi China; 2 Department of Ultrasound, The First Affiliated Hospital of Xi’an Jiaotong University, 277 Yanta Western Road, Xi’an, 710061 Shaanxi China

**Keywords:** Breast neoplasms, Growth, Prognosis, Public health

## Abstract

**Background:**

To date, anatomic tumor length is a key criterion for cancer staging and can be used to evaluate the effectiveness of therapies. This article describes growth pattern that can be used as a new characteristic to represent disease burden and tumor features and predict lymphatic metastasis.

**Methods:**

Patients with breast cancer were included in this 10-year (1999–2008) hospital-based multicenter retrospective study. The pathologic length/height ratio was used to illustrate the correlation between tumor features, behaviors and treatments in breast malignancies. The most appropriate ratio was chosen based on the comprehensive evaluation of *p* value and changing trend of each characteristic.

**Results:**

The sample consisted of 4211 women diagnosed with breast cancer. Among them, 2037 patients with complete pathologic length, width and height information were included in the final analysis. There were 2.34 ± 4.77 metastatic lymph nodes for spheroid tumors and 3.21 ± 5.82 for ellipsoid tumors when the cutoff point was 2. In addition, the proportion of ellipsoidal tumors gradually increased from 54.36 to 56.67% in the upper outer quadrant (UOQ) and from 6.7 to 9.03% in the central region with an increase in the cutoff point. The proportion of ER + PR + ellipsoid tumors significantly decreased from 50.1 to 45.35% and that of ER–PR ellipsoid tumors significantly increased from 32.73 to 36.24% with an increase in the cutoff point. Additionally, the best length/weight ratio to distinguish spheroid and ellipsoid tumors was 2.

**Conclusion:**

This study described for the first time how growth pattern is correlated with tumor malignancy and how it influences the selection of therapeutic strategies for patients.

**Electronic supplementary material:**

The online version of this article (10.1007/s12282-019-01041-7) contains supplementary material, which is available to authorized users.

## Introduction

Breast cancer is the most prevalent malignancy in women and the second most common cause of cancer-related death worldwide [1, 2]. The accumulation of cancer cells can be reflected by the spatial shape of the tumor. The formation of a solid mass is the result of constant interactions between proliferating cancer cells and their micro-environment. The cells of solid tumors initiate the process of invasion and metastasis along their spatial growth that can ultimately lead to fatal distant diseases. In women with breast cancer, up to 30% node-negative cases and 70% of node-positive cases will recur [3].

The anatomic TNM classification provides a common language for communicating disease burden. Tumor size is a key criterion for cancer staging and can be used to evaluate the effectiveness of therapies [4]. However, *T* only describes the largest tumor diameter of the tumor and provides limited description of tumor features despite each tumor having a unique three-dimensional shape. In particular, Wapnir et al. demonstrated that there is a significant overestimation of tumor volume when using the greatest diameter alone for early breast cancer cases [5]. Biological behaviors vary in invasive breast tumors and should not be neglected even though molecular biology is currently widely used in disease staging and prognostic prediction. A larger tumor size, a greater number of metastatic lymph nodes, and distant metastasis represent a later stage. For tumors sharing the same immunophenotype and histologic type, T3 or T4 stage tumors display a rather indolent behavior without rapidly progressing or metastasizing, while some T1 stage tumors have been found to be highly proliferate and metastatic with extremely aggressive behavior. This compels us to investigate whether other tumor features may be associated with tumor behaviors. The morphological growth pattern of tumors is one characteristic that can vary, whereby some tumors display a relative spheroid growth pattern, while others have an ellipsoid growth pattern. Whether the morphological growth pattern can be incorporated with other clinical characteristics is still largely unknown.

In this study, tumors were divided into spheroid and ellipsoid groups based on pathologic length, width and height. We observed a relationship between growth pattern and age, side, primary tumor quadrant, number of lesions, metastatic lymph node number, histologic type, hormone receptor expression, HER-2 expression and treatment. We aimed to investigate whether growth patterns affect tumor features and behaviors and treatment decision making.

## Materials and methods

### Data and patients

Our data were obtained from the Nationwide Multicenter 10-year (1999–2008) Retrospective Clinical Epidemiological Study of Breast Cancer in China. The study was led by Cancer Hospital/Institute, Chinese Academy of Medical Sciences (CICAMS), and seven Grade Three A hospitals nationwide were included.

The details of region and hospital selection have been previously described. Seven geographic regions that cover most areas and represent different levels of breast cancer burden were selected, including the North, Northeast, Central, South, East, Northwest, and Southwest regions. The selected Grade Three A hospitals were some of the best leading regional public hospitals and referral centers. The source of the inpatients was able to cover the corresponding research area. Information on pathologic analysis, diagnosis, surgery, radiotherapy, medical oncology, and routine follow-up care for breast cancer patients was provided. The Zhang group has proven and demonstrated that this sampling can represent the basic characteristics of breast cancer in China in particular [6].

### Data collection and quality control

One month was randomly selected from each year between 1999 and 2008 for the collection of inpatient data from each hospital. January and February were excluded from the random selection to avoid the influence of the biggest annual Chinese holiday. At least 50 pathologically diagnosed inpatient cases from the selected month each year were enrolled in the review. If the number of available cases was lower than 50 in a selected month, the cases from 2 months immediately before and after the selected month were included.

As previously described, the following data were systematically collected for all enrolled patients via medical chart review: (1) general information; (2) demographic characteristics at the time of diagnosis/admission; (3) breast cancer risk factors; (4) results of clinical breast examination (CBE); (5) results of diagnostic imaging; (6) use of currently available surgical approaches, radiotherapy, chemotherapy and molecular targeted therapy for breast cancer; and (7) pathologic characteristics. Pathological examinations included the detection of ER, PR and HER-2 expression by immunohistochemistry (IHC). IHC analysis was conducted prior to the study. ER+ or PR+ cases were defined as hormone receptor positive. For HER-2, cases without staining in the cytomembrane of cancer cells was defined as HER-2-. Any proportions of cancer cells exhibiting weak or incomplete cytomembrane staining or < 10% of cancer cells exhibiting weak or complete cytomembrane staining were defined as HER-2+; ≥ 10% of cancer cells exhibiting weak or moderately intact cytomembrane staining or < 10% cancer cells exhibiting uniform, strong and complete cytomembrane staining were defined as HER-2++; ≥ 10% of cancer cells exhibiting consistent, strong and intact cytomembrane staining were defined as HER-2+++; HER-2- or HER-2+ was considered as HER-2 low expression, which was considered negative in clinical therapy; HER-2+++ was considered HER-2 positive; and HER-2++required further confirmation with fluorescent in situ hybridization (FISH). If no gene expression was observed in the FISH analysis, HER-2 was categorized as negative; otherwise, it was positive.

All of the patients’ information was collected by standard case report forms (CRFs) designed by the Cancer Hospital/Institute, Chinese Academy of Medical Sciences (CICAMS). Data on general information, risk factors, diagnostic imaging tests, therapy models, and pathologic characteristics were independently entered twice from the paper to a computer-based database by two well-trained clerks from each hospital. The data were transmitted to CICAMS and verified by EpiData (https://www.epidata.dk/). After inconsistency and logical errors were corrected, the revised database was sent back to CICAMS for final analysis. Specific details were described in our previous study [6].

### Patient and variable selection

A total of 4211 patients with breast cancer were included in the study. Due to the lack of some of the tumor pathologic width and height data, only data from 2037 of 4211 patients who had complete tumor pathologic information of length, width and height were analyzed in this paper. Age at diagnosis was considered a continuous variable, side of tumor, primary tumor location [categorized into upper inner quadrant (UIQ), upper outer quadrant (UOQ), lower inner quadrant (LIQ), lower outer quadrant (LOQ), and central], pathological type (categorized into ductal carcinoma in situ with microinvasion (DCIS-Mi), invasive ductal carcinoma (IDC), invasive lobular carcinoma (ILC), tubular carcinoma, mucinous carcinoma, medullary carcinoma and other types of invasive carcinoma), expression of estrogen receptor (ER), progesterone receptor (PR), and human epidermal growth factor receptor-2 (HER-2) (categorized as positive and negative), treatment (categorized into excision of breast mass, non-conserving surgery and conserving surgery), and metastatic LN numbers were recorded according to H&E and IHC exams.

### Definition

Three diameters of the tumors were provided in pathologic data: the longest axis was defined as the length, the shortest axis was defined as the height, and the axis in the middle was defined as the width. The ratio was defined as length/height and was used to measure the growth pattern. The typical figures of the tumor growth patterns (spheroid and ellipsoid groups) were shown in the supplementary Fig. 1. The mean length/height was 1.73, and we set up 3 length/height cutoff points (1.5, 1.73, and 2) to describe eccentricity to varying degrees. For example, if the ratio is < 2 when the cutoff point is 2, the growth pattern would be more spheroid. ER and PR were considered positive if immunostaining was positive in more than 1% of tumor cells. HER-2 positivity was defined by a score of 3+ on IHC or amplification on FISH.

### Statistical analysis

The mean ± SD was calculated to describe continuous variables, and a constituent ratio was used to describe categorical variables. *T* tests were used for the comparison of continuous variables, and the Chi-square test was used for comparison of categorical variables.

All statistical analyses were performed using SPSS (version 19.0, Chicago, IL, USA) and GraphPad Prism (version 7, GraphPad Software Inc., California, USA). A two-tailed *p* value < 0.05 was considered statistically significant.

### Ethics statement

This study was approved by the Institutional Review Board of the Cancer Foundation of China. Because of the retrospective nature of the study, we were unable to contact all patients or their families. In addition, informed consent was not obtained because this study was not a risk to patients. All patient identifiers were removed, as per the approved procedures. Deidentified data were saved in a secured database, to which only researchers in our teams had access.

## Results

### General characteristics of 2037 breast cancer cases

**Fig. 1:**
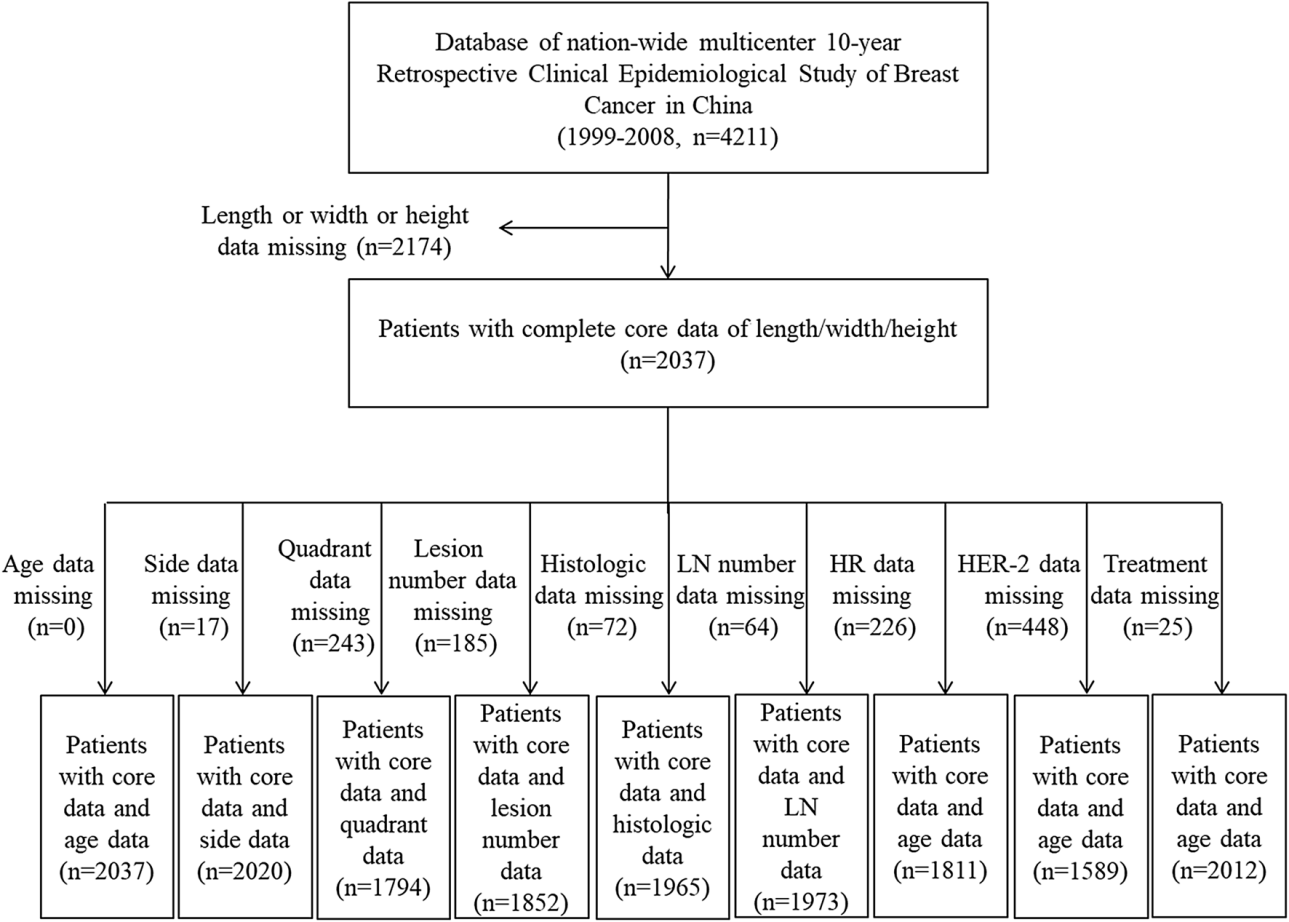
A schematic representation of how the data were included or excluded. *HR* hormone receptor, *LN* lymph nodes, *ER* estrogen receptor, *PR* progesterone receptor, *HER-2* human epidermal growth factor receptor-2

A total of 4211 eligible breast cancer patients were enrolled in this study. We excluded 2174 patients because they lacked complete 3-D tumor size data in their pathologic reports. A total of 2037 patients (48.37%) with complete 3-D tumor size data were ultimately included in this study. Table 1 summarizes the demographic and clinicopathological characteristics of all enrolled patients. Figure 1 shows a flow diagram of data processing.

**Table 1:** Characteristics of participants who completed the analysis

Characteristics	Number of cases %	
Age at diagnosis		
* N*	2037	
Side		
*N*	2020	
Left	1074	53.17
Right	946	46.83
Primary tumor quadrant		
*N*	1794	
UIQ	336	18.73
UOQ	972	54.18
LIQ	135	7.53
LOQ	227	12.65
Central	124	6.91
Numbers of lesions		
*N*	1852	
1	1784	96.33
2	68	3.67
Histologic type		
*N*	1965	
DCIS-Mi	61	3.10
IDC	1765	89.82
ILC	55	2.80
Medullary carcinoma	39	1.98
Mucinous carcinoma	28	1.42
Others	17	0.87
Pathologic LN numbers	1973	
Hormone receptor status		
* N*	1811	
ER (+) PR (+)	920	50.80
ER (+) PR (−)	157	8.67
ER (−) PR (+)	169	9.33
ER (−) PR (−)	565	31.20
T stage		
*N*	2037	
T1	710	34.86
T2	1067	52.38
T3	260	12.76
N stage		
*N*	1973	
N0	988	50.08
N1	570	28.89
N2	243	12.32
N3	172	8.72
M stage		
*N*	2037	
M0	1982	97.30
M1	55	2.70
HER-2 status		
*N*	1589	
Positive	528	33.23
Negative	1061	66.77
Treatment		
*N*	2012	
Excision of breast mass	23	1.14
Conserving	107	5.32
Non-conserving	1882	93.54

*UIQ* upper inner quadrant, *UOQ* upper outer quadrant, *LIQ* lower inner quadrant, *LOQ* lower outer quadrant, *DCIS-Mi* ductal carcinoma in situ with micro-invasion, *IDC* invasive ductal carcinoma, *ILC* invasive lobular carcinoma, *LN* lymph nodes, *ER* estrogen receptor, *PR* progesterone receptor, *HER-2* human epidermal growth factor receptor-2

### Comparison of clinical characteristics among groups with different growth patterns

**Fig. 2:**
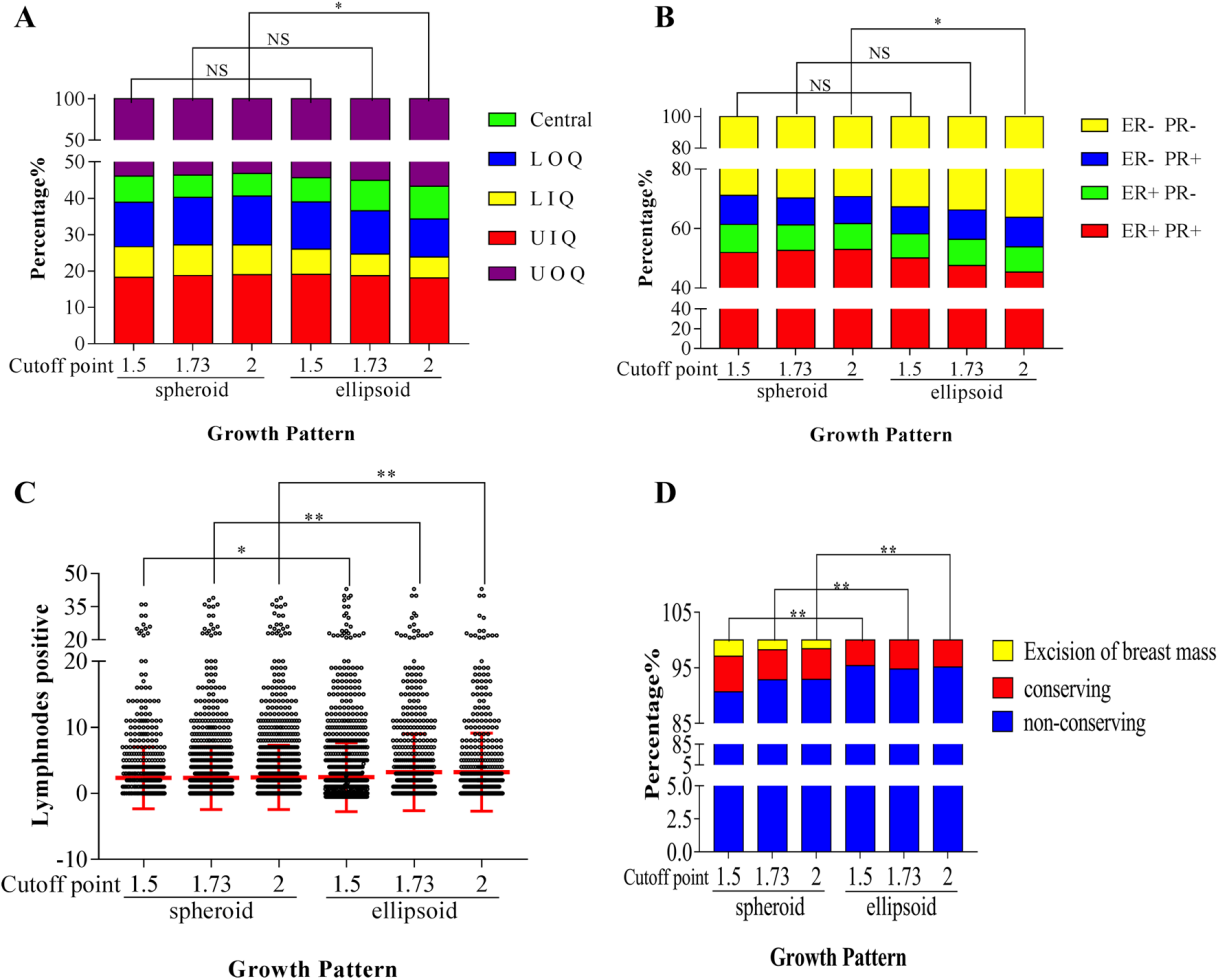
The trends and changes in hormone receptor, quadrant, lymph node metastasis and treatment with different cutoff points. **a** The proportion of each hormone receptor status according to the two growth patterns at different cutoff points. **b** The proportion of different quadrants according to the two growth patterns at different cutoff points. **c** Metastatic lymph nodes according to the two growth patterns at different cutoff points. **d** The proportion of different treatments according to the two growth patterns at different cutoff points. *p < 0.05, **p < 0.01, NS *p* ≥ 0.5. *UIQ* upper inner quadrant, *UOQ* upper outer quadrant, *LIQ* lower inner quadrant, *LOQ* lower outer quadrant, *ER* estrogen receptor, *PR* progesterone receptor, *HER-2* human epidermal growth factor receptor-2

For the primary tumor quadrant, the *p* value gradually decreased from 0.81 (cutoff point 1.5) (Table 2) to 0.032 (cutoff point 2) (Table 4), with a statistically significant difference between the spheroid and ellipsoid groups at the cutoff point of 2. In addition, the proportions of ellipsoidal tumors gradually increased from 54.36 to 56.67% in the UOQ and 6.7 to 9.03% in the central region (Fig. 2a). The percentage distribution indicated that ellipsoid tumors are more likely to be present in the UOQ and central region. Our data also showed that age at diagnosis was significantly different (*t* = 2.28, *p* = 0.023) only when the cutoff point was 1.5 (Table 2), and we considered these negative data because further significant differences were not observed. The side of the tumor did not influence the growth pattern of breast tumors in 3 classifications.

**Table 2:** Comparison of characteristics between different growth patterns at cutoff point of 1.5

Characteristics	Spheroid		Ellipsoid		*p* value
	number	%	number	%	
Age at diagnosis	808		1229		0.023^a^
	50.15 ± 11.00		48.97 ± 10.42		
Side	800		1220		0.739^b^
Left	429	53.63	645	52.87	
Right	371	46.37	575	47.13	
Primary tumor quadrant	705		1089		0.810^b^
UIQ	129	18.30	207	19.01	
UOQ	380	53.90	592	54.36	
LIQ	59	8.37	76	6.98	
LOQ	86	12.20	141	12.95	
Central	51	7.23	73	6.70	
Numbers of lesions	1153		699		0.000^b^
1	1127	97.75	657	93.99	
2	26	2.25	42	6.01	
Histologic type	764		1201		0.311^b^
DCIS-Mi	21	2.75	40	3.33	
IDC	679	88.87	1086	90.42	
ILC	26	3.40	29	2.41	
Medullary carcinoma	19	2.49	20	1.67	
Mucinous carcinoma	14	1.83	14	1.17	
Others	5	0.65	12	1.00	
Pathological LN numbers	761		1212		0.037^a^
	2.35 ± 4.67		2.85 ± 5.50		
Hormone receptor status	705		1106		0.308^b^
ER (+)PR (+)	366	51.91	554	50.09	
ER (+)PR (−)	67	9.50	90	8.14	
ER (−)PR (+)	69	9.79	100	9.04	
ER (−)PR (−)	203	28.79	362	32.73	
HER-2 status	619		970		0.717^b^
Positive	209	33.76	319	32.89	
Negative	410	66.24	651	67.11	
Treatment	794		1218		0.000^b^
Excision of breast mass	23	2.90	0	0.00	
Conserving	51	6.42	56	4.60	
Non-conserving	720	90.68	1162	95.40	

*UIQ* upper inner quadrant, *UOQ* upper outer quadrant, *LIQ* lower inner quadrant, *LOQ* lower outer quadrant, *DCIS-Mi* ductal carcinoma in situ with micro-invasion, *IDC* invasive ductal carcinoma, *ILC* invasive lobular carcinoma, *LN* lymph nodes, *ER* estrogen receptor, *PR* progesterone receptor, *HER-2* human epidermal growth factor receptor-2

^a^The *p* value of *t* test

^b^The *p* value of Chi-square test

### Comparison of pathological characteristics among groups with different growth patterns

We analyzed the number of tumors and pathologically confirmed metastatic lymph nodes, as these factors may be affected by the growth pattern of breast cancer. We found that the number of average metastatic lymph nodes was 2.85 in ellipsoid tumors vs 2.35 in spheroid tumors at the cutoff point of 1.5 (Table 2), 3.21 in ellipsoid tumors vs 2.34 in spheroid tumors at the cutoff point of 1.73 (Table 3), and 3.22 in ellipsoid tumors vs 2.44 in spheroid tumors at the cutoff point of 2 (Table 4). Although the *p* value did not strictly increase gradually based on the cutoff point, the average number of metastases was significantly increased to 1.73 vs 1.5 (Fig. 2c). This trend showed that patients with ellipsoid tumors were more susceptible to lymph node metastasis. Although a significant difference was found in the numbers of tumors at the cutoff point of 1.5 (Table 2) and ellipsoidal tumors were more likely to have 2 lesions in pathologic reports, we failed to see a positive result at the cutoff point of 1.73 (Table 3) or 2 (Table 4). In our study, no difference in pathologic type was found among the 3 cutoff points.

**Table 3:** Comparison of characteristics between different growth patterns at cutoff point of 1.73

Characteristics	Spheroid		Ellipsoid		*P* value
	number	%	number	%	
Age at diagnosis	1300		737		0.271^a^
	49.62 ± 10.77		49.11 ± 10.47		
Side	1290		730		0.870^b^
Left	691	53.76	383	51.61	
Right	599	46.24	347	48.39	
Primary tumor quadrant	1146		648		0.155^b^
UIQ	215	18.76	121	18.67	
UOQ	615	53.66	357	55.09	
LIQ	96	8.38	39	6.02	
LOQ	150	13.09	77	11.88	
Central	70	6.11	54	8.33	
Numbers of lesions	1150		700		0.280^b^
1	1112	96.70	670	95.71	
2	38	3.30	30	4.29	
Histologic type	1240		725		0.095^b^
DCIS-Mi	30	2.13	31	5.59	
IDC	1114	79.01	651	117.30	
ILC	36	2.55	19	3.42	
Medullary carcinoma	30	2.13	9	1.62	
Mucinous carcinoma	20	1.42	8	1.44	
Others	10	0.71	7	1.26	
Pathological LN numbers	1245		728		0.000^a^
	2.34 ± 4.77		3.21 ± 5.82		
Hormone receptor status	1153		658		0.202^b^
ER (+) PR (+)	607	52.65	313	47.57	
ER (+) PR (−)	99	8.59	58	8.81	
ER (−) PR (+)	104	9.02	65	9.88	
ER (−) PR (−)	343	29.75	222	33.74	
HER-2 status	1020		569		0.988^b^
Positive	334	32.75	194	34.09	
Negative	686	67.25	375	65.91	
Treatment	1283		729		0.001^b^
Excision of breast mass	23	1.79	0	0.00	
Conserving	69	5.38	38	5.21	
Non-conserving	1191	92.83	691	94.79	

*UIQ* upper inner quadrant, *UOQ* upper outer quadrant, *LIQ* lower inner quadrant, *LOQ* lower outer quadrant, *DCIS-Mi* ductal carcinoma in situ with micro-invasion, *IDC* invasive ductal carcinoma, *ILC* invasive lobular carcinoma, *LN* lymph nodes, *ER* estrogen receptor, *PR* progesterone receptor, *HER-2* human epidermal growth factor receptor-2

^a^The *p* value of *t* test

^b^The *p* value of Chi-square test

**Table 4:** Comparison of characteristics between different growth patterns at cutoff point of 2

Characteristics	Spheroid		Ellipsoid		*P* value
	number	%	number	%	
Age at diagnosis	1472		565		0.450^a^
	49.15 ± 10.49		49.55 ± 10.73		
Side	1462		558		0.387^b^
Left	786	53.76	288	51.61	
Right	676	46.24	270	48.39	
Primary tumor quadrant	1307		487		0.032^b^
UIQ	248	18.97	88	18.07	
UOQ	696	53.25	276	56.67	
LIQ	107	8.19	28	5.75	
LOQ	176	13.47	51	10.47	
Central	80	6.12	44	9.03	
Numbers of lesions	1312		538		0.090^b^
1	1270	96.80	512	95.17	
2	42	3.20	26	4.83	
Histologic type	1410		555		0.207^b^
DCIS-Mi	36	2.55	25	4.50	
IDC	1269	90.00	496	89.37	
ILC	42	2.98	13	2.34	
Medullary carcinoma	31	2.20	8	1.44	
Mucinous carcinoma	21	1.49	7	1.26	
Others	11	0.78	6	1.08	
Pathological LN numbers	1414		559		0.003^a^
	2.44 ± 4.87		3.22 ± 5.91		
Hormone receptor status	1306		505		0.018^b^
ER (+)PR (+)	691	52.91	229	45.35	
ER (+)PR (−)	114	8.73	43	8.51	
ER (−)PR (+)	119	9.11	50	9.90	
ER (−)PR (−)	382	29.25	183	36.24	
HER-2 status	1153		436		0.584^b^
Positive	383	33.22	145	33.26	
Negative	770	66.78	291	66.74	
Treatment	1454		558		0.009^b^
Excision of breast mass	23	1.58	0	0.00	
Conserving	80	5.50	27	4.84	
Non-conserving	1351	92.92	531	95.16	

*UIQ* upper inner quadrant, *UOQ* upper outer quadrant, *LIQ* lower inner quadrant, *LOQ* lower outer quadrant, *DCIS-Mi* ductal carcinoma in situ with micro-invasion, *IDC* invasive ductal carcinoma, *ILC* invasive lobular carcinoma, *LN* lymph nodes, *ER* estrogen receptor, *PR* progesterone receptor, *HER-2* human epidermal growth factor receptor-2

^a^The *p* value of *t* test

^b^The *p* value of Chi-square test

### Comparison of molecular subtypes among groups with different growth patterns

HER-2 status and HR status were analyzed separately. A gradually decreasing *p* value from 0.308 at a cutoff point of 1.5–0.018 at a cutoff point of 2 was observed regarding the HR status. As shown in Fig. 2b, the proportions of 4 subtypes according to HR status (ER+ PR+, ER+ PR−, ER-PR+, and ER-PR−) of spheroid tumors did not change much, while the proportion of ER+PR+ ellipsoid tumors significantly decreased from 50.1 to 45.35% and that of ER–PR ellipsoid tumors significantly increased from 32.73 to 36.24% with the increase in cutoff point. This result indicated that HR-negative breast cancer cases were more likely to exhibit ellipsoid growth than HR-positive breast cancer cases. There were no significant differences in the subgroups between the two HER-2 statuses.

### Comparison of treatment among groups with different growth patterns

Apart from objective differences, growth pattern also affected the doctors’ decisions for treatment. Irrespective of the cutoff point, compared with ellipsoid tumors, spheroid tumors showed significant differences in surgery type (Tables 2, 3 and 4 and Fig. 2d). 23 of 2037 patients received excision of breast mass only in local hospitals instead of standard breast conserving surgery in these 7 Grade 3A hospitals/institutes, and all of these patients had spheroid tumors. Meanwhile, patients who underwent breast-conserving surgery were more likely to have spheroid tumors.

## Discussion

In this study, we preliminarily investigated the association between growth patterns and tumor features, tumor behavior and surgery decision making. Our approach provides new insights into tumor features, as we focused on identifying a new way to distinguish breast cancer by dividing growth patterns into spheroids and ellipsoids. The length/height ratio can partially reflect how the masses progressed and help us to infer the possible dynamic nature of the tumor. In particular, we chose postoperative pathologic data (length, width, height, HR status, and HER-2 status) to make our analysis more accurate and reliable. This is because preoperative imaging data have more measurement error when calculating the true size of tumors, and preoperative biopsy samples of a small tumor region may not enable the representative characterization of the tumor as a whole.

Lymph node metastasis acts as a very strong prognostic indicator in breast cancer. In this study, the results showed that an ellipsoid growth pattern has greater potential to cause lymph node metastasis. More metastasis further indicates a worse prognosis. Consistent with our study, Han et al. had shown that tumors with high eccentricity result in worse survival outcomes due to the high incidence of unfavorable prognostic factors, their results also showed that there was a significant association between the degree of eccentricity and the time to distant metastasis in patients with hormone receptor-negative tumors [7]. Our results showed that HR-positive tumors had a more spheroid shape than other subtypes, while the HER-2 status alone had no influence on the growth pattern. This result is in conflict with breast imaging studies that showed triple-negative tumors having more round shapes and smooth margins [8]. We think our finding is more reliable mainly because of two reasons: (1) pathologic data are more accurate than imaging results. (2) There were 176 patients in their study, and the number of patients in their study was small. Therefore, the statistical significance of their data may be insufficient. We also observed that ellipsoid masses are more likely to be located in the central or UOQ of the breast. The reasons for the increase in ellipsoid tumors in the UOQ of the breast remain unknown at this time. This may be due to the development of genetic alterations and more blood supply from axillary in that region. We have also explored the relationship between growth patterns and several closely related breast cancer factors, such as patient age, tumor side, number of lesions and pathologic classification. The lack of meaningful significance prompted us to explore whether the growth pattern has an impact on treatment decision making. Our data showed that the rate of breast-conserving surgery was significantly reduced in patients with ellipsoid masses. Breast-conserving surgery in China often relies on the incision margin of intraoperative frozen pathology. Chinese surgeons prefer to remove a larger area to ensure a clear margin. For ellipsoid tumors, more excised tissue based on length is not conducive to maintain the breast shape, less excised tissue based on height is not conducive to clear margin. Thus, spheroid tumors are more conducive to the extent of resection and margin control. There were 23 patients whose breast masses had already been removed in other small hospitals when they came to these 7 hospitals. Surgical margin was not reported, because small hospitals do not perform intraoperative frozen pathology. They were reluctant to receive further standard breast-conserving surgery due to poor economic conditions or aging. All of the 23 patients’ cancer types were luminal A/B without high-risk factors. Tumors were considered less malignant and can be well controlled by endocrine therapy. Interestingly, all patients who only received mass removal as treatment had spheroid tumors. This phenomenon was also consistent with our data that HR-positive breast cancer cases were more likely to exhibit spheroid growth. We had also tracked the survival status of all patients in our single center. The total number of patients whom we could track with the follow-up was 483. We divided them into four groups according to the molecular subtypes (HR+/HER2−; HR+/HER2+; HR−/HER2+; HR−/HER2−). The survival curves could be seen in supplementary Fig. 2 and 3. The survival analysis also showed that the ellipsoid-like HR+/HER2− and HR−/HER2+ tumors were more likely to have bad RFS and OS.

We explored 3 cutoff points to characterize the growth pattern to find the best criterion. Although the age of patients and number of lesions showed significant differences when the length/height was 1.5, we failed to observe a further positive *p* value when the ratio was 1.73 or 2. Taking all the characteristics into consideration, we would prefer to set the cutoff point at 2. We also tested the ROC curves in different ratios including 1.5, 1.73 and 2. The supplementary Fig. 4 showed that when the cutoff point was 2, the model could best reflect the prognostic value of breast cancer patients based on the lymph node status. When the tumor length is more than double the height, the tumor may have more lymph node metastasis and may be more likely to be HR negative and located in the UOQ or central part of the breast. In addition, patients who receive breast-conserving surgery are more likely to have spheroid tumors.

We believe that the growth pattern of tumors is not random, but determined by the expression of certain genes. It was interesting to notice that increased IL-6 secretion and activation of STAT3 were observed in a Ras-transformed subculture of mammary epithelial cells in 3D cell culure, a phenotype also seen in xenografts and human tumors, but not in standard 2D cultures [9]. Han's team had found that 54 genes were significantly correlated with tumor eccentricity in breast cancer. Among the genes with the highest *p* value, some genes related to extracellular matrix remodeling such as MMP13 and adamts12 were related to the tumors’ growth pattern [7]. Besides, breast cancer lesions are not homogeneous. Evidence of intratumor heterogeneity has been discovered by chromosomal- and microarray-based comparative genomic hybridization (CGH) and massively parallel sequencing (MPS) analyses [10, 11]. According to the clonal evolution model, intratumoral heterogeneity includes differences in their genetic, phenotypic or behavioral characteristics [10–12]. In individual breast cancer cases, different subclonal populations of cancer cells may exist across different geographical regions of a tumor (spatial heterogeneity) or evolve over time between the primary tumor and a subsequent local or distant recurrence (temporal heterogeneity) [13, 14]. We hypothesized that different growth patterns may be one of the visible manifestations of genetic heterogeneity. Similar with breast cancer, colorectal tumors which have a laterally spreading pattern show unique expression features of various genes including β-catenin, type IV collagen, and PKC [15]. If highly proliferative clones are located in one marginal area, they are likely to form ellipsoid tumors and cause more metastasis. This finding also supported the idea that a small proportion of cancer cells located at the invading front of solid tumors can determine local invasion and metastasis, while the remaining tumor cells remain nonmetastatic [16].

There are some limitations in our analysis as follows: (i) we did not explore any mechanism regarding why the growth pattern is associated with tumor location, hormone receptor status or number of metastatic lymph nodes. (ii) Although postoperative specimens were used, the tumor size was measured after formalin fixation. Hsu et al. reported that formalin fixation may cause tumor shrinkage and that the measurement after formalin fixation results in an underestimation of actual tumor size [17]. (iii) The complex contour of the malignant mass can be radial and cannot be fully characterized by the dimensional diameter alone. (iv) The concept of TNBC subtypes was first proposed in China in 2019 [18], but our data were obtained from 1999–2008 when the Chinese doctors did not have advanced knowledge of triple negative breast cancer subtypes. The relationship between tumor growth pattern and tumor malignancy in each subtype of TNBC was not included in the analysis. Further study is still necessary to elucidate the mechanism of emergence of different shapes.

## Conclusions

In the present study, we performed simple categorization of breast cancer patients based on tumor growth patterns. Our findings indicate that the tumor growth pattern can be a useful indicator to predict tumor malignancy and the prognostic value of breast cancer and that it can contribute to the selection of an optimal therapeutic strategy for individual patients.

## Availability of data and materials

The data generated during this study are included in this article. Raw data are available upon reasonable request.

## Electronic supplementary material

Below is the link to the electronic supplementary material.

**Supplementary figure 1.** Typical figures of the tumor growth patterns (A: spheroid ; B: ellipsoid groups). (TIF 1213 kb)

**Supplementary figure 2.** The relationship between growth pattern and recurrent-free survival (RFS) in our single center. (TIF 4466 kb)

**Supplementary figure 3.** The relationship between growth pattern and overall survival (OS) in our single center. (TIF 4456 kb)

**Supplementary figure 4.** The ROC curves based on the lymph nodes status. (JPG 25 kb)

## Abbreviations

HR: Hormone receptor
LN: Lymph nodes
UIQ: Upper inner quadrant
UOQ: Upper outer quadrant
LIQ: Lower inner quadrant
LOQ: Lower outer quadrant
ER: Estrogen receptor
PR: Progesterone receptor
HER-2: Human epidermal growth factor receptor-2
