# Supplementary figures and images for: Growth pattern can be used as a new characteristic to predict malignancy in breast cancer
Source: Breast Cancer. 2020 Jan 3;27(3):445–55. doi: 10.1007/s12282-019-01041-7 (PMC7196087; doi:10.1007/s12282-019-01041-7)

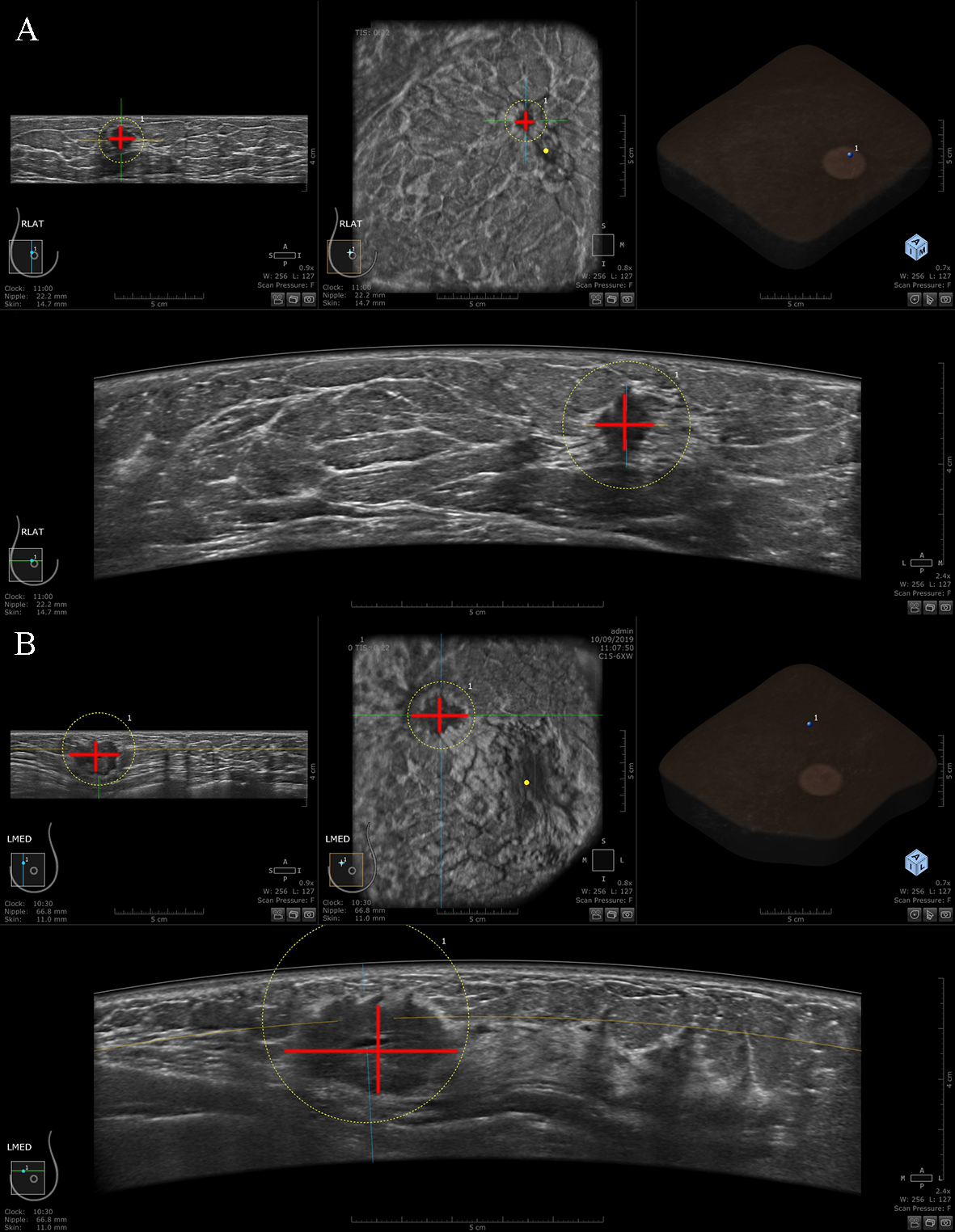

Supplement: Supplementary file 1 — Supplementary figure 1. Typical figures of the tumor growth patterns (A: spheroid ; B: ellipsoid groups). (TIF 1213 kb) [file 12282_2019_1041_MOESM1_ESM.tif]

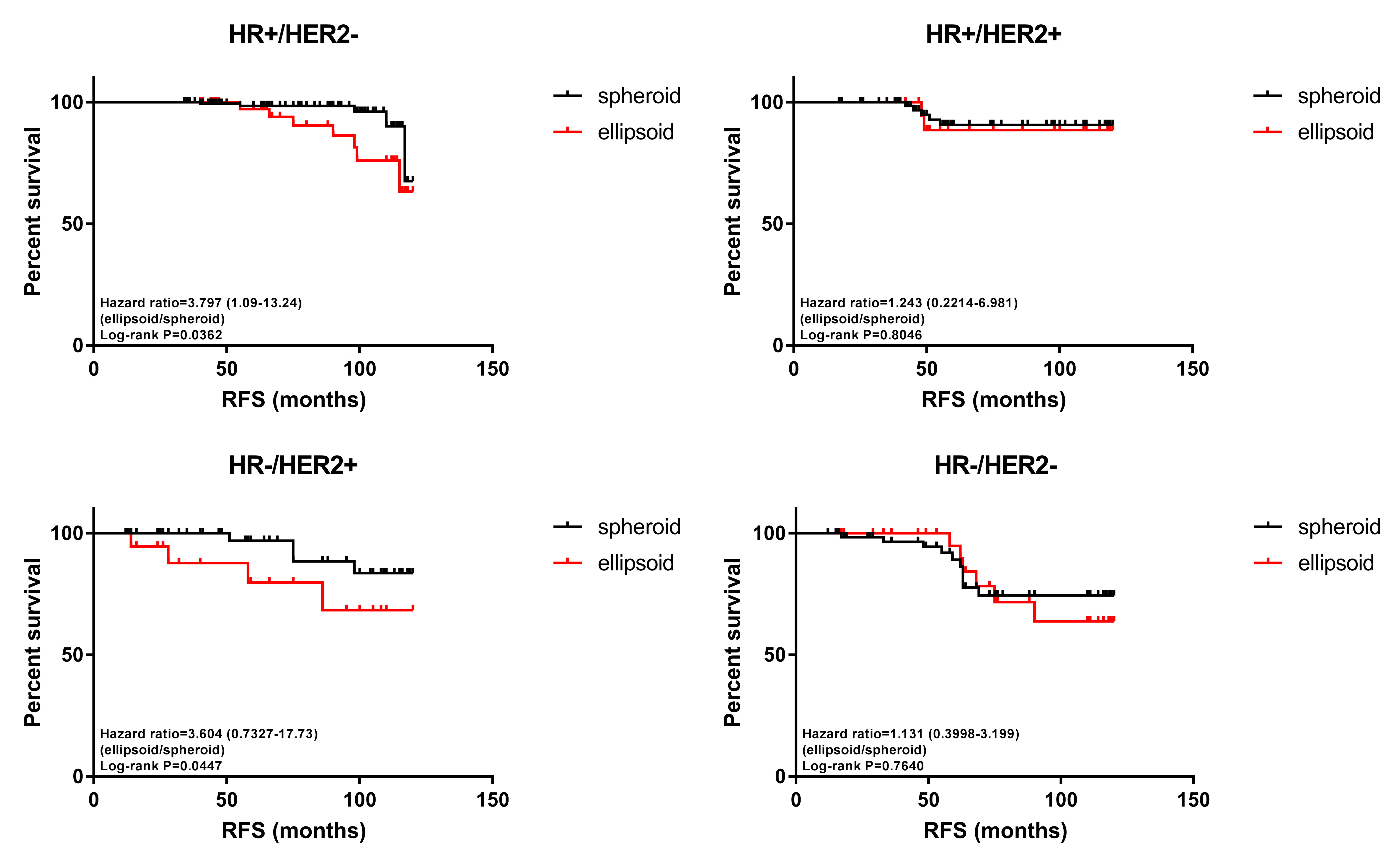

Supplement: Supplementary file 2 — Supplementary figure 2. The relationship between growth pattern and recurrent-free survival (RFS) in our single center. (TIF 4466 kb) [file 12282_2019_1041_MOESM2_ESM.tif]

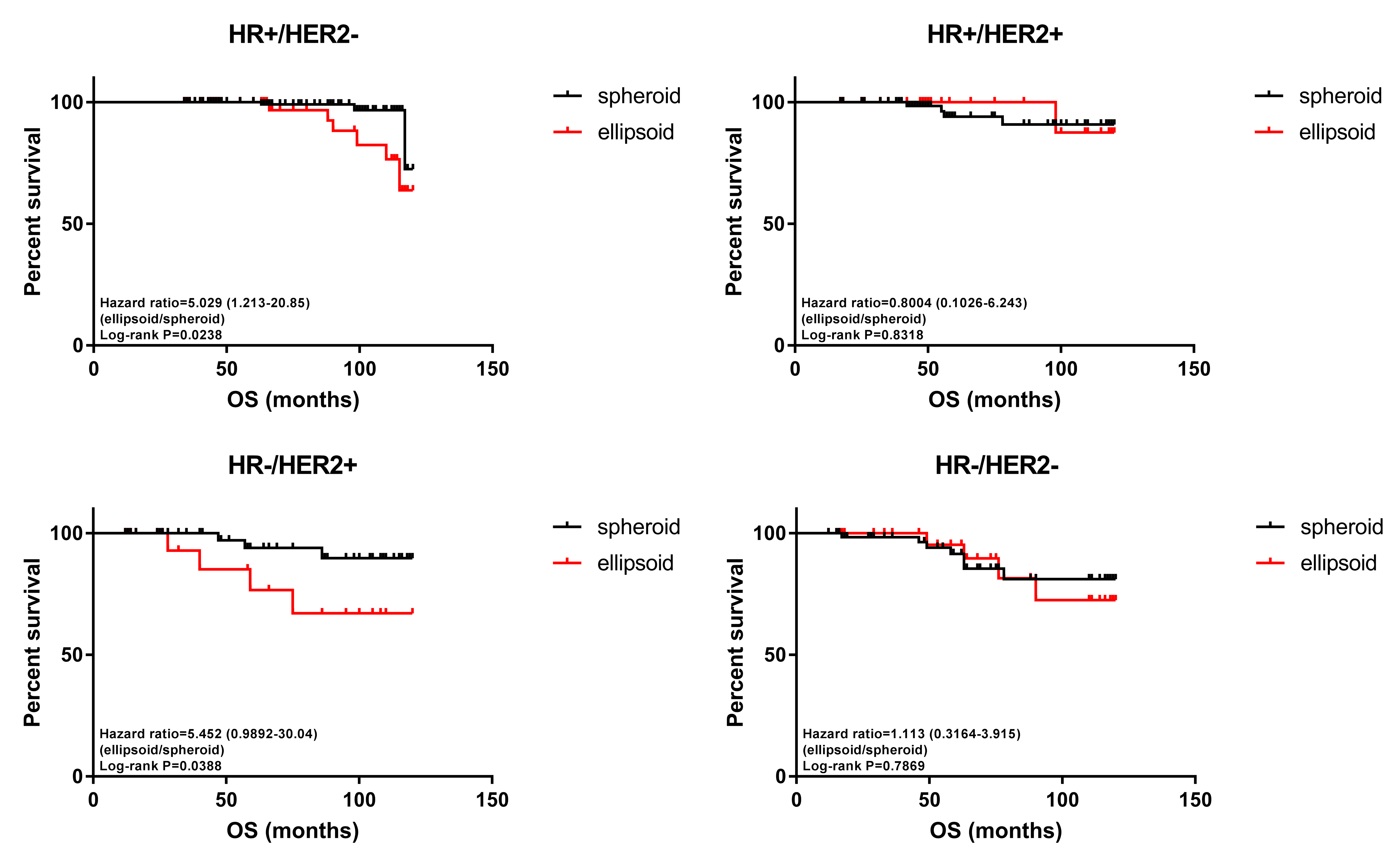

Supplement: Supplementary file 3 — Supplementary figure 3. The relationship between growth pattern and overall survival (OS) in our single center. (TIF 4456 kb) [file 12282_2019_1041_MOESM3_ESM.tif]

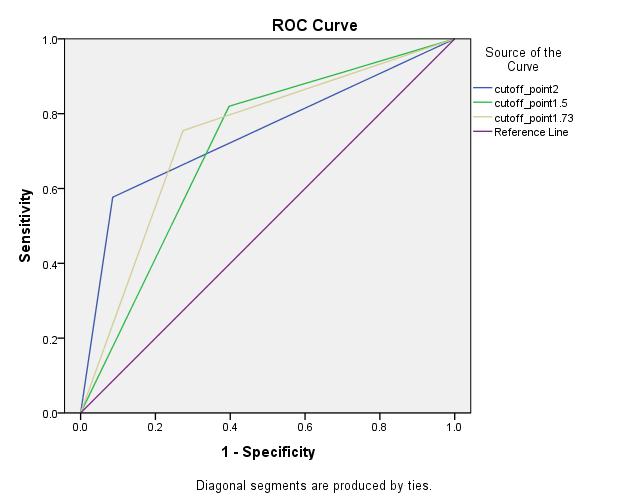

Supplement: Supplementary file 4 — Supplementary figure 4. The ROC curves based on the lymph nodes status. (JPG 25 kb) [file 12282_2019_1041_MOESM4_ESM.jpg]
